# Supplementary material for: The Association of Procalcitonin and C-Reactive Protein with Bacterial Infections Acquired during Intensive Care Unit Stay in COVID-19 Critically Ill Patients
Source: Antibiotics (Basel). 2023 Oct 12;12(10):1536. doi: 10.3390/antibiotics12101536 (PMC10604665; doi:10.3390/antibiotics12101536)
Supplement: Supplementary file 1 [file antibiotics-12-01536-s001.zip › antibiotics-2619142-supplementary.pdf]

Table S1. CRP and PCT values at and after infection diagnosis.

	All Patients (*n* = 279)	No Infection (*n* = 110)	Yes Infection (*n* = 169)	p Value
**PCT D −1 (ng/mL; median, IQR)**	0.1 (0.1–0.3)	0.1 (0.1–0.1)	0.2 (0.1–0.4)	<0.001
**PCT D +0 (ng/mL; median, IQR)**	0.2 (0.1–0.6)	0.1 (0.1–0.1)	0.3 (0.1–1.0)	<0.001
**PCT D +1 (ng/mL; median, IQR)**	0.3 (0.1–0.9)	0.1 (0.1–0.2)	0.4 (0.2–1.2)	<0.001
**CRP D −1 (mg/dL; median, IQR)**	1.2 (0.1–4.7)	0.4 (0.1–2.1)	2.1 (0.5–7.2)	<0.001
**CRP D +0 (mg/dL; median, IQR)**	1.8 (0.3–5.8)	0.4 (0.1–2.2)	3.2 (1.2–12.8)	<0.001
**CRP D +1 (mg/dL; median, IQR)**	1.6 (0.1–6.2)	0.2 (0.1–1.2)	3.9 (1.1–16.7)	<0.001

D -1: Day before infection or day 10 after ICU admission; D +0: day of infection diagnosis or day 11 after ICU admission, D + 1 the day after diagnosis of infection or day 12 after ICU admission.

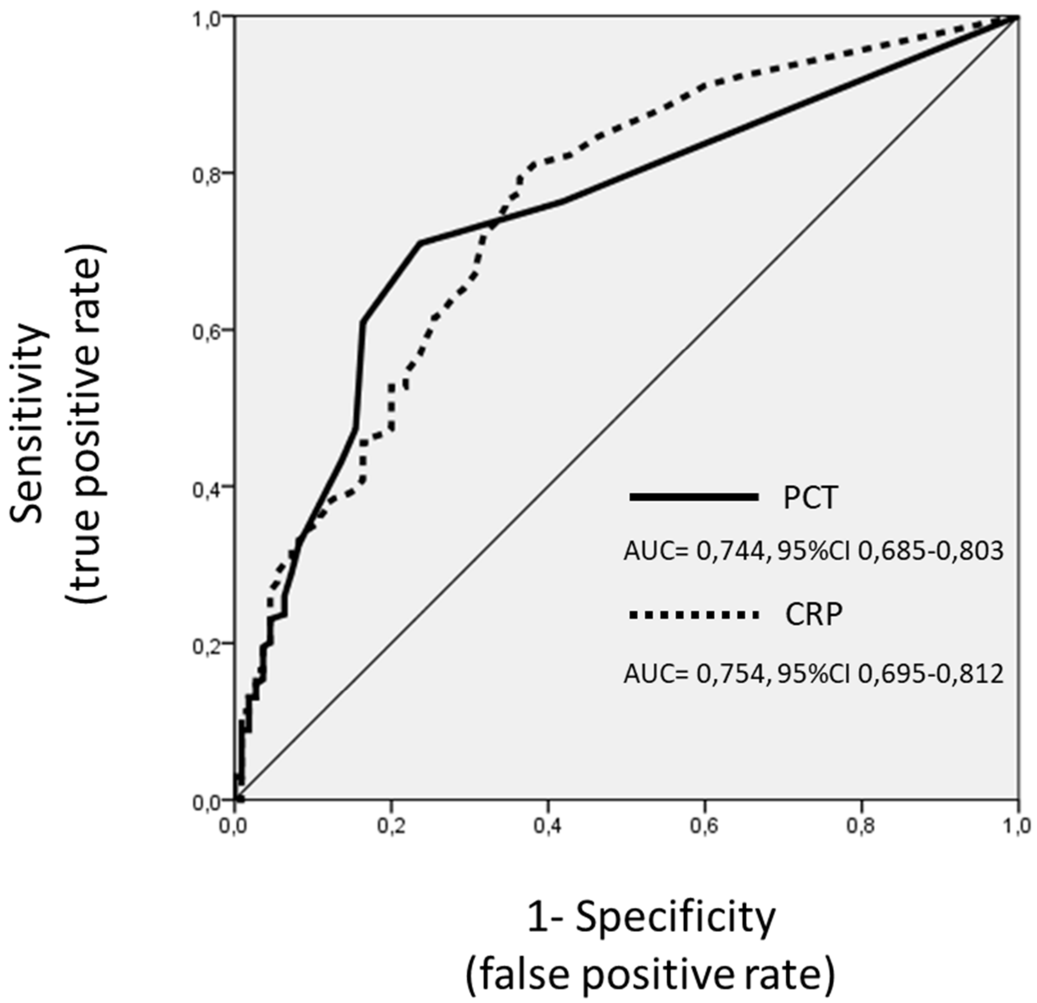

Figure S1. Receiver operating characteristic curve for PCT (solid line) and CRP (dotted line).

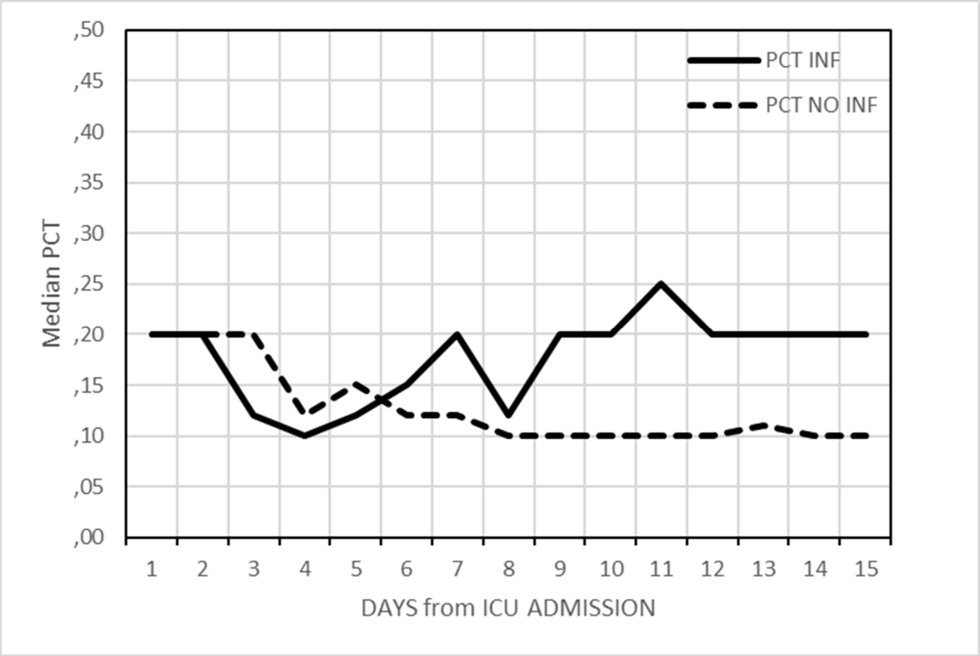

Figure S2. Median PCTvalues in patients with and without infections acquired in ICU in the first 14 days after ICU admission.

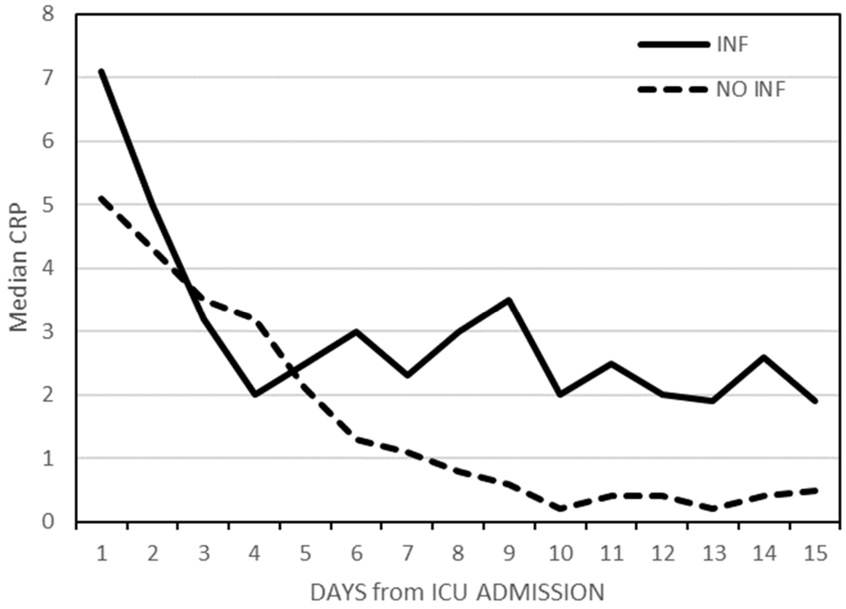

Figure S3. Median CRP values in patients with and without infections acquired in ICU in the first 14 days after ICU admission.
